# Antibiotic Resistance Determinant-Focused *Acinetobacter baumannii* Vaccine Designed Using Reverse Vaccinology

**DOI:** 10.3390/ijms18020458

**Published:** 2017-02-21

**Authors:** Zhaohui Ni, Yan Chen, Edison Ong, Yongqun He

**Affiliations:** 1 Department of Pathogenobiology, College of Basic Medical Science, Jilin University, Changchun 130021, China; nich@jlu.edu.cn; 2 Unit for Laboratory Animal Medicine, Department of Microbiology and Immunology, University of Michigan, Ann Arbor, MI 48109, USA; edong@umich.edu; 3 Department of Neurosurgery, The Second Hospital of Jilin University, Changchun 130041, China; drchenyan@jlu.edu.cn; 4 Department of Computational Medicine and Bioinformatics, University of Michigan, Ann Arbor, MI 48109, USA; 5 Center of Computational Medicine and Bioinformatics, University of Michigan, Ann Arbor, MI 48109, USA

**Keywords:** *Acinetobacter baumannii*, reverse vaccinology, Vaxign, antibiotic resistance, vaccine candidate, bioinformatics

## Abstract

As one of the most influential and troublesome human pathogens, *Acinetobacter baumannii* (*A. baumannii*) has emerged with many multidrug-resistant strains. After collecting 33 complete *A. baumannii* genomes and 84 representative antibiotic resistance determinants, we used the Vaxign reverse vaccinology approach to predict classical type vaccine candidates against *A. baumannii* infections and new type vaccine candidates against antibiotic resistance. Our genome analysis identified 35 outer membrane or extracellular adhesins that are conserved among all 33 genomes, have no human protein homology, and have less than 2 transmembrane helices. These 35 antigens include 11 TonB dependent receptors, 8 porins, 7 efflux pump proteins, and 2 fimbrial proteins (FilF and CAM87009.1). CAM86003.1 was predicted to be an adhesin outer membrane protein absent from 3 antibiotic-sensitive strains and conserved in 21 antibiotic-resistant strains. Feasible anti-resistance vaccine candidates also include one extracellular protein (QnrA), 3 RND type outer membrane efflux pump proteins, and 3 CTX-M type β-lactamases. Among 39 β-lactamases, *A. baumannii* CTX-M-2, -5, and -43 enzymes are predicted as adhesins and better vaccine candidates than other β-lactamases to induce preventive immunity and enhance antibiotic treatments. This report represents the first reverse vaccinology study to systematically predict vaccine antigen candidates against antibiotic resistance for a microbial pathogen.

## 1. Introduction

*Acinetobacter baumannii* is a Gram-negative opportunistic bacterial pathogen that is responsible for a diverse range of infections including ventilator-associated pneumonia, skin and wound infections, urinary tract infections, meningitis and bacteremia [1]. The majority of infections caused by *A. baumannii* are hospital-acquired, most commonly in the intensive care setting of severely ill patients. In addition, severe community-acquired pneumonia caused by *A. baumannii* has also been reported [2]. *A. baumannii* has become one of the most important and troublesome human pathogens with its increased number of infections and emergence of more threatening multidrug-resistant and pan-drug resistant strains [3]. Antibiotic resistance has greatly affected the effectiveness of antibiotic treatments. The development of alternative approaches is necessary.

Vaccination strategies are emerging as a viable option to prevent and/or treat multi- or pan- drug-resistant infections. Although there is currently no licensed vaccine against *A. baumannii* infections, various vaccine candidates, including inactivated whole cell [4], outer membrane complexes (OMCs) [5], outer membrane vesicles (OMVs) [6], OmpA [7], Ata [8], Bap [9], K1 capsular polysaccharide [10] and PNAG [11], have recently been proven effective at some levels to protect against challenges in animals (mostly mice) from homologue strains and clonally distinct clinical isolates through active or passive immunization strategies. However, the clinical applications of reported vaccine candidates are limited by the potential regulatory and safety, and these antigens do not directly target antibiotic resistance [12]. β-lactamases are the most prevalent mechanism of antibiotic resistance in *A. baumannii* [13]. Ciofu et al. showed over 10 years ago that the animals immunized with β-lactamase proteins could induce strong neutralizing antibody responses and significant lower bacterial load and pathology [14,15]. This suggests an important solution against antibiotic resistance. However, no further progress has been reported along this line of research. Therefore, identification of more vaccine antigens that stimulate best immune responses against infections as well as antibiotic resistance is still a major task and challenge.

As an emerging and revolutionary vaccine development approach, reverse vaccinology (RV) starts with the prediction of vaccine protein targets by bioinformatics analysis of genome protein-coding sequences [16]. With the initial bioinformatics analysis, RV facilitates rapid vaccine design with less reliance on conventional animal testing and clinical trials. RV has been applied to the development of vaccines against a variety of pathogens such as serogroup B *Neisseria meningitidis* (MenB) [17], *Bacillus anthracis* [18], *Streptococcus pneumoniae* [19], *Mycobacterium tuberculosis* [20], and *Cryptosporidium hominis* [21]. More recently, several laboratories around the world began to identify *A. baumannii* vaccines candidates by RV approach [22,23,24,25]. Using 10 complete and 31 draft *A. baumannii* genomes, Moriel1 et al. applied RV and identified 42 *A. baumannii* antigens as potential vaccine targets [25]. By applying RV to analyze 14 *A. baumannii* genome sequences, Chiang et al. identified 13 novel proteins as potential vaccine candidates, and 3 out of these 13 antigens (OmpK, FKBP-type 22KD peptidyl-prolyl cis-trans isomerase (FKIB), Ompp1) were experimentally tested and proven to be highly immunogenic and conferred partial protection (60%) in a mouse pneumonia animal model [23]. Hassan et al. recently estimated the pan-genome of 30 complete *A. baumannii* genome sequences and identified 13 highly antigenic proteins as conserved immunogenic targets [24]. These three RV studies focused on the identification of conserved outer membrane and secreted proteins. The study conducted by Hassan et al. [24] also analyzed other aspects including transmembrane helices, 3D protein structural analysis, epitope mapping, protein function analysis. Out of 57 vaccine candidates predicted by Vaxign RV analysis using *A. baumannii* ATCC19606 genome sequences, Singh et al. specifically studied an outer membrane protein FilF and confirmed its value as a protective antigen [22]. However, no report is available on the usage of RV for vaccine design against *A. baumannii* antibiotic resistance.

Vaxign is the first web-based vaccine design program that predicts vaccine targets based on the RV strategy [26,27,28]. The Vaxign computational pipeline includes the prediction of many features, including subcellular localization, topology (transmembrane helices and β barrel structure), adhesin probability, sequence similarity to other pathogen sequences, similarity to host (e.g., human) genome sequences, and MHC class I and II epitope predictions [27]. In addition to the *A. baumannii* genome analysis by Singh et al. [22], the Vaxign RV approach has successfully predicted vaccine targets for many pathogens such as uropathogenic *Escherichia coli* [26], *Brucella spp*. [29], *Rickettsia prowazekii* [30], *Streptococcus agalactiae* [31], *Corynebacterium pseudotuberculosis* [32], and *Campylobacter* [33]. However, how Vaxign can be used for predicting antibiotic resistance determinants as vaccine candidates has not been demonstrated.

In this study, we used Vaxign and other bioinformatics methods to analyze 33 complete genome sequences and 84 representative antibiotic resistance determinants to predict *A. baumannii* protective vaccine antigens with a focused effort to identify antibiotic resistance determinants feasible for anti-resistance vaccine development.

## 2. Results

### 2.1. Collection of A. baumannii Genome Sequences and Resistance Genes

In this study, we used 33 completed and annotated genome sequences from National Center for Biotechnology Information (NCBI) database, which consist of both multi-drug resistant (MDR) strains and sensitive strains and represent the main epidemic *A. baumannii* lineages spread worldwide. The information of these strains including strain names, NCBI BioProject numbers, the years and locations of isolation, antibiotic resistance, and associated diseases are summarized in Table 1. Among these 33 strains, strains ATCC17978, AB307-0294, and D1279779 are sensitive to antibiotics [23,34,35], and other strains are resistant to multiple antibiotics drugs.

In addition, our study collected 84 representative resistance genes from the ARDB-Antibiotic Resistance Genes Database (Available online: https://ardb.cbcb.umd.edu/), and NCBI Gene and Protein databases. Figure S1 provides the protein accession numbers and Vaxign analysis results for all these resistance genes. Extracted from various *A. baumannii* strains (e.g., *A. baumannii* AYE, ACICU, AB0057), these genes encode for proteins responsible for the resistance to most classes of antibiotics used in clinical practice, including β-lactams, aminoglycosides, quinolones, chloramphenicols, tetracyclines, sulfanilamide, tremethoprimand and polymycin. These resistance determinants utilize various antimicrobial resistance mechanisms, including enzymatic mechanisms, changes in OMPs and multidrug efflux pumps (Table 2).

**Figure 1:**
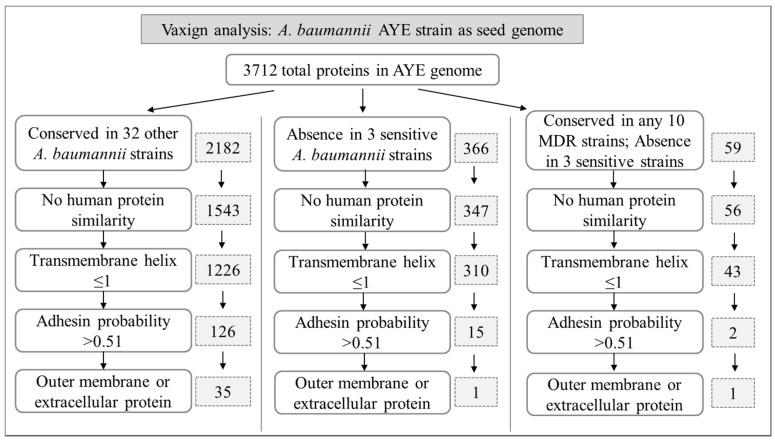
Vaxign analysis pipelines and results.

The above 33 complete genome sequences and 84 resistance determinants sequences were used to perform Vaxign RV analysis to identify *A. baumannii* vaccine candidate antigens. For the resistance determinants analysis, all 84 antibiotic resistance proteins were combined for a one single Vaxign pipeline analysis. Our overall RV prediction pipelines and results are shown in Figure 1 and are detailed in the following sections.

### 2.2. Predicted A. baumannii Vaccine Targets Based on RV Analysis of 33 Genomes

*A. baumannii* strain AYE is a representative multidrug-resistant strain that is epidemic in France [36]. Containing a resistance genomic island with 45 resistance genes clustered, this strain has been frequently used in whole genome sequence analyses (including RV analyses) [23,25,36,37]. The proteome of strain AYE includes 3712 proteins. In our Vaxign analysis, strain AYE was also used as the reference seed strain. As shown in Figure 1 and detailed below, Vaxign generated different results given different parameter settings.

#### 2.2.1. Predicted *A. baumannii* Vaccine Targets Conserved in All 33 Genomes

Among all 3712 proteins of strain AYE, 2182 are conserved among all 33 strains. A BLASTp sequence comparison found that 639 of these 2182 proteins have homology with human proteins. Pathogen antigens having holomogy with host proteins are not preferred vaccine candidates since they likely induce autoimmunity or immune tolerance [38]. After removing these proteins and proteins with >1 transmembrane helices, we obtained 126 adhesin proteins. Proteins with a transmembrane domain less than or equal to one are selected since multiple transmembrane domains makes the purification of recombinant proteins difficult [38]. Adhesins are important for bacterial invasion and infection and are preferred vaccine candidates [38,39,40,41]. Outer membrane proteins (OMPs) and extracellular proteins are more likely to be protective vaccine candidates able to stimulate strong protective immunity [38,42]. Out of 126 adhesin proteins, 35 were predicted to be outer membrane or extracellular proteins (Figure 1).

Table 3 provides more detailed information about these 35 proteins. Specifically, these proteins include 27 OMPs and 8 extracellular proteins. The adhesin probabilities of these 35 proteins are between 0.513 to 0.744, which are all above 0.51, the cutoff of assigning a protein as an adhesin [43]. These 35 proteins have the lengths between 143 to 855 amino acids (Table 3).

To refine the selection, the antigenicity scores calculated by the VaxiJen v2.0 server (Available online: http://www.ddg-pharmfac.net/vaxijen/VaxiJen/VaxiJen) were applied. Our Vaxijen analysis found that all the 35 potential vaccine candidates have antigenicity scores from 0.42 to 0.82. An antigenicity score of over 0.40 is an indication of protein antigenicity [44]. Therefore, all 35 candidates were predicted to be antigenic.

For Cluster of Orthologous Groups (COG) protein functional categorization, 33 out of the 35 proteins fall into 7 different functional groups. Among these groups, “cell wall/membrane/envelope biogenesis” and “inorganic ion transport and metabolism” have 11 proteins each. Four COG groups have one protein each, including “secondary metabolites biosynthesis, transport, and catabolism”, “posttranslational modification, protein turnover, chaperones”, “cell motility, intracellular trafficking, secretion, and vesicular transport”, and “cell wall/membrane/envelope biogenesis, inorganic ion transport and metabolism”, and “function unknown” group has 7 proteins (Table 3).

Domains are distinct functional and/or structural units of proteins. A conserved domain footprint may reveal aspects of a protein’s molecular or cellular function [45]. Of the total 35 potential vaccine candidates, 11 proteins were identified as TonB dependent receptors (PF00593), and most of them are putative ferric siderophore receptor proteins. An example TonB dependent receptor is CAM86022.1 (baumannii acinetobactin utilization, BauA), which has been identified to play an important role in iron uptake [46]. Another large group of proteins is defined as porin or efflux related proteins. Eight porin proteins, such as OmpA family lipoprotein (CAM87753.1) and OprD superfamily porins proteins (CAM88440.1 and CAM86576.1), were identified. Seven efflux related proteins, such as ATP binding cassette (ABC) superfamily transporter (CAM85361.1) and resistance nodulation cell division (RND) family transporter (CAM85249.1 and CAM85825.1), were predicted by Vaxign. In addition, fimbrial or pilus related protein (CAM87009.1 and CAM87933.1), copper resistance protein NlpE (CAM87612.1) and some other proteins with known or unknown functions were also identified. The detailed information is summarized in Table 3.

#### 2.2.2. Predicted *A. baumannii* Vaccine Targets Absent from 3 Antibiotic-Sensitive Strains

Among the 33 *A. baumannii* strains, three strains (i.e., AB307-0294, ATCC17978, and D1279779) are drug sensitive (Table 1). We hypothesized that those drug resistance determinants commonly found in MDR strains most likely do not exist in these drug sensitive strains. When we excluded any proteins in these three drug sensitive strains, 366 proteins were found in our Vaxign analysis (Figure 1). These 366 proteins include many commonly known drug resistance determinants such as β-lactamases, aminoglycoside modifying enzymes, dihydrofolate reductase, chloramphenicol resistance protein, tetracycline resistance proteins and an efflux transporter. After applying no human similarity and transmembrane helix criteria, Vaxign predicted 15 adhesins. Fourteen of these 15 adhesins include many hypothetic proteins and one drug resistance determinant chloramphenicol acetyltransferase (CAM88367.1). Note that CAM88367.1 was predicted to have an unknown subcellular location (probability = 0.2). Interestingly, only one of these 15 adhesins exists in outer membrane or extracellular locations. Specifically, this protein is CAM86003.1, a 390 amino acid protein with an adhesin probability of 0.655 and an outer membrane location (probability = 0.952). This protein has been annotated as “conserved hypothetic protein; putative signal peptide” (Available online: https://www.ncbi.nlm.nih.gov/protein/CAM86003.1).

In addition to the absence from three drug sensitive strains, we added a criterion that a protein needed to be conserved in at least 10 other strains. With these two restrictions, Vaxign found 59 proteins from strain AYE (Figure 1). After using the criteria of no human similarity and transmembrane helix ≤1, we found 2 adhesins. These two adhesins include CAM86003.1 (as described above) and CAM86739.1. CAM86739.1 is another hypothetical protein with only 96 amino acids and an unknown function. Further analysis found that CAM86003.1 is conserved in 21 antibiotic-resistant strains, and CAM86739.1 is conserved in 19 antibiotic-resistant strains. These results suggest that these two proteins are possible protective antigens worth experimental evaluations.

### 2.3. Predicted A. baumannii Vaccine Targets Based on RV Analysis of 84 Antibiotic Resistance Determinants

Subcellular localization is a major selection criterion in RV analysis. To target against antibiotic resistance, we hypothesize that human antibody response plays a major role. Therefore, outer membrane and extracellular antibiotic resistance determinants, esp. those with high adhesin probabilities, are most favorable vaccine candidates against antibiotic resistance. Owing to their capability of hydrolyzing most found β-lactam antibiotics, β-lactamases form the most prevalent mechanism to β-lactam resistance. Typically produced in bacterial periplasms, β-lactamases can be packed inside outer membrane vesicles (OMVs) which can be released to the extracellular environment. The released β-Lactamase-containing OMVs then induce the production of anti-β-lactamse IgG [47,48]. In addition, *Pseudomonas aeruginosa* β-lactamase proteins have been found to induce strong neutralizing antibody responses and lower bacterial load in animal models [14,15]. Therefore, for the prediction of *A. baumannii* vaccine targets against antibiotic resistance, we also included those resistance determinants with periplasmic localization.

Among 84 resistance proteins, 1 extracellular protein, 2 OMPs and 21 periplasmic proteins were identified. The single predicted extracellular protein is QnrA (ADB64519.1), which is responsible for quinolone resistance. The two predicted OMPs are adeC/adeK/oprM family multidrug efflux complex outermembrane factor (WP_000045119.1) and one hypothetical protein (WP_000018327.1). The 21 predicted periplasmic proteins are all β-lactamases, including TEM, SHV, CTX-M, VEB, RTG and ADC. Of these 24 resistance proteins, 4 proteins have an adhesin probability of >0.51. Among these 4 proteins, 3 are CTX-M-type extended-spectrum β-lactamases (ESBLs), and one is WP_000018327.1. The antigenicity prediction showed that except for ADC, the other 23 candidates had an antigenic score > 0.4, indicating that they were antigenic.

Among 84 representative resistance determinants, 39 are β-lactamases, and 21 of these β-lactamases are predicted to be periplasmic (Table 4). β-lactamases can be classified into Ambler class A to D, including class A extended-spectrum β-lactamases (ESBLs), class B metallo-β-lactamases (MBLs), class C Acinetobacter-derived cephalosporinases (ADCs), and carbapenem-hydrolyzing class D β-lactamases (CHDLs). The 21 β-lactamases include one class C ADC β-lactamase and 20 class A β-lactamases. Previous β-lactamase vaccine studies used class C AmpC β-lactamase [14,15]. However, AmpC β-lactamase may not be the only or the best vaccine antigen. Our Vaxign RV analysis of 39 β-lactamases found that 3 class A CTX-M-type ESBLs, including CTX-M-2, -5, and -43, all have adhesin scores > 0.51, which is the cutoff for defining an adhesin [43]. No β-lactamase in class C was found to have such a feature.

**Figure 2:**
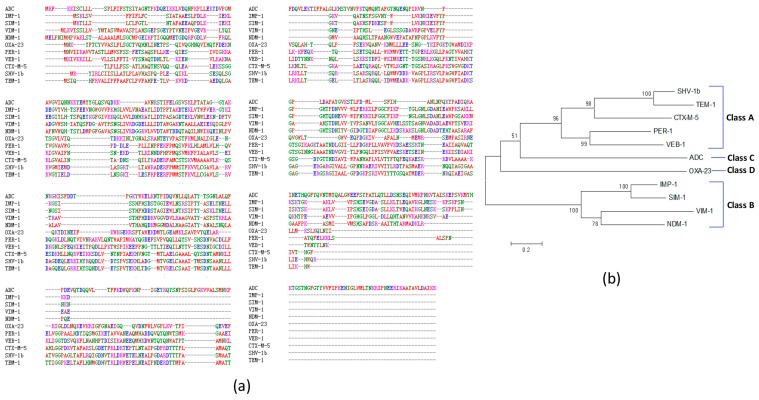
Multiple protein sequence alignment and phylogenetic tree analysis of 11 representative β-lactamases. (**a**) Multiple sequence alignment using Clustal Omega. Different colors indicate the following: red: residue AVFPMILW; blue: residue DE; magenta: residue RK; green: residue STYHCNGQ; grey: others. The sign “–“(dash) means no amino acid aligned; (**b**) Phylogenetic tree analysis of these 11 β-lactamases. MEGA 6.0 Neighbor-Joining method with 500 bootstraps and standard settings was applied. The tree is drawn to scale. Branch lengths are in the same units as those of the evolutionary distances used to infer the phylogenetic tree. The numbers shown in the tree are the percentages of replicate trees in which the associated taxa clustered together in the bootstrap test (500 replicates).

Our further multiple sequence alignment identified many sequence gaps exist among 11 representative β-lactamases (Figure 2a). The protein sequence identity among these 11 β-lactamases ranges from 11.64% to 66.26%, indicating significant sequence variations among β-lactamases. Among Ambler class A β-lactamases (TEM, SHV, CTX-M, PER and VEB), TEM-1 shares extensive similarity with SHV-1 β-lactamases (65.49%). The amino acid sequence similarities of CTX-M-5 with TEM-1 and SHV-1 are 35.04% and 37.09%, respectively. Among Ambler class B metal β-lactamases (IMP, SIM, VIM and NDM), IMP-1 and SIM-1 share the highest similarity (66.26%). However, Ambler class C (ADC) and Ambler class D (OXA) β-lactamases possess fewer identities with other β-lactamases (<20%).

Our phylogenetic tree analysis further shows the evolutionary relationships and distances among these 11 proteins (Figure 2b). Four classes of β-lactamases were separately clustered. Specifically, 5 class A β-lactamases were clustered together, 4 class B β-lactamases were grouped in its own cluster. The one class C and one class D β-lactamases were separated from the class A or B clusters.

## 3. Discussion

The major contributions of this study are two-fold. First, we applied RV methods to analyze 33 complete *A. baumannii* genomes and identify many *A. baumannii* vaccine candidates using traditional whole genome analysis methods. Second, our RV methods with these 33 genomes and an additional collection of 84 representative antibiotic resistance determinants identified many vaccine candidates against antibiotic resistance. The second contribution also represents the first study to use an RV strategy for systematic vaccine design against antibiotic resistance for any microbial pathogen.

A very significant finding from our study is the list of 11 TonB-dependent receptors among all 35 predicted vaccine candidates conserved among 33 genomes (Table 1). The RV studies by Moriel1 et al. [25] and Chiang et al. [23] did not identify any such receptor, and Hassan et al. [24] identified only one such receptor. TonB-dependent receptors are bacterial OMPs that primarily bind and transport ferric chelates called siderophores and also support the transport of vitamin B12, nickel, and carbohydrates in a TonB-dependent manner [49]. Iron transport into the cytosol is mediated by these specific TonB-dependent membrane receptors that recognize the iron-siderophore complexes. Iron acquisition is generally required for bacterial growth during infection [50]. The expression of a siderophore-ferric complex receptor is also critical for the establishment and persistence of *A. baumannii* infections [51]. It has been found that antibodies directed against proteins associated with iron uptake exert a bacteriostatic or bactericidal effect against *A. baumannii* in vitro [52]. Recently, the recombinant BauA, a member of TonB-dependent receptors, was experimentally verified as a valid protective vaccine antigen as shown by mouse challenge and passive serum immunization experiments [46]. Interestingly, surface-exposed iron receptors have also been used in the development of vaccines against other human pathogens e.g., uropathogenic *E. coli* [53]. Our identification of 11 TonB-dependent receptors provides more options of using TonB-dependent receptors in *A. baumannii* vaccine development. It would also be important to determine how these different TonB dependent receptors interact with each other.

Our RV analysis identified a putative pilus assembly protein (FilF) (CAM87933.1) and a fimbrial protein CAM87009.1. Recombinant FilF protein was experimentally verified to elicit a strong protective response against *A. baumannii* [22]. Fimbriae are proteinaceous filaments expressed on the surfaces of many pathogenic bacteria. These filaments are involved in bacterial adhesion to the host cells and are considered important for virulence [32]. Fimbriae are recognized as potential vaccine antigens of several pathogenic bacteria e.g., *E. coli* [33] and *Bordetella pertussis* [34]. Given its high adhesin probability, our study predicted the important role of CAM87009.1 in fimbrial adhesion. The role of CAM87009.1 as a protective antigen deserves experimental evaluation.

Porins are proteins that are able to form channels allowing the transport of hydrophilic compounds up to a certain size exclusion limit across lipid bilayer membranes [54,55]. Porins can play a variety of roles depending on the bacterial species, including transport of small molecules, maintenance of cellular structural integrity, bacterial conjugation and bacteriophage binding. Porins may also play a significant role in antibiotic resistance [56]. The porin protein OmpA is the most abundant surface protein and plays a role in the permeability of small solutes such as β-lactams and saccharides. In addition, OmpA of *A. baumannii* serves as an important virulence factor in the bacterial interaction with epithelial cells, induction of apoptosis of host cells, and dissemination of bacteria into the bloodstream [57]. OmpA has been identified as a highly promising candidate for active and passive immunization based on humoral immunodominance during lethal *A. baumannii* infection in mice [7]. Using an immunoproteome-based approach, Fajardo et al. also demonstrated that porin-related proteins including OmpA and OprB-like are highly abundant on the bacterial surface and can mount an immune response [56]. In our study, eight porin-related proteins including OmpA family lipoproteins (CAM87753.1), OprD superfamily porin proteins (CAM88440.1 and CAM86576.1), OprB family porin proteins (CAM85599.1, CAM86576.1), and Porin_7 superfamily proteins (CAM85174.1 and CAM85154.1) were identified. This list not only includes known vaccine candidates OmpA and OprB but also other porins whose potential roles as protective antigens and virulence factors are still unknown and are worth experimental testing.

Considering that OmpA is very conserved in the outer membranes of Gram-negative bacteria, it might be possible to develop a universal vaccine using OmpA against various bacterial pathogens. Chen et al. [58] compared the homology of OmpA proteins in three pathogenic *Yersinia* and found that their amino acid sequences have >98.6% identity. Cross-immunogenicity was also observed using three pathogenic *Yersinia* OmpAs [58], further supporting the speculation that OmpA is likely to be a common protective antigen against pathogenic *Yersinia* [58]. We performed a multiple amino acid sequence alignment analysis with 10 OmpA amino acid sequences from ten different bacteria, including 8 bacteria from the family *Enterobacteriaceae* (i.e., *Escherichia coli*, *Klebsiella pneumonia*, *Serratia marcescens*, *Yersinia bercovieri*, *Proteus mirabilis*, *Salmonella enterica subsp. enterica serovar Typhimurium*, *Shigella flexneri*, and *Cronobacter sakazakii*), *Brucella abortus*, and *A. baumannii.* Our study indicates that although the OmpA proteins from these 8 *Enterobacteriaceae* bacteria share high identities (70.06% to 97.38%), the sequence identities between *A. baumannii* (or *B. abortus*) and these 8 bacteria are only 21%–29% (data not shown). Therefore, an OmpA vaccine against many bacteria in one species or one family can likely be developed. However, it may be difficult to develop a universal OmpA vaccine against all bacteria in different families. Meanwhile, a possible effect of an OmpA vaccine on microbiota may need to be investigated if such an OmpA vaccine against many bacteria is developed.

Efflux pumps are transport proteins involved in the extrusion of toxic substrates (including antibiotics of multiple classes) from within cells into the external environment, leading to the prevention of intracellular accumulation of toxic compounds [59]. The overexpression of these transporters is associated with bacterial multidrug resistance [55]. Five superfamilies of efflux systems are found in *A. baumannii*: ATP-binding cassette (ABC) transporters, resistance-nodulation-cell division (RND) family, small multidrug resistance (SMR), multidrug and toxic compound extrusion (MATE) families, and the major facilitator superfamily (MFS) [60]. Our study found one ABC transporter, i.e., MlaD protein (CAM85361.1), which is a toluene tolerance efflux transporter. MlaD is predicted to be secreted to the extracellular environment and has high adhesin probability and antigenicity, suggesting its potential role as a protective vaccine antigen. Overproduction of RND efflux pumps has been found to be associated with MDR, virulence, and biofilm formation in *A. baumannii* [61]. Our genome sequence analysis found two RND transporters CAM85249.1 (CzcC) and CAM85825.1. Based on our BLAST sequence analysis, the CzcC sequence is assigned with NCBI reference sequence ID WP_005115306.1, which is a conserved RND transporter among over 100 *A. baumannii* strains (Available online: https://www.ncbi.nlm.nih.gov/protein/WP_005115306.1). These two proteins are outer membrane proteins with high adhesin probability and antigenicity scores, suggesting their potential usage in vaccine development against *A. baumannii* infections. Considering their role in MDR, these two proteins are possible candidates for developing vaccines against drug resistance (see more discussion below).

Recent studies have identified the potential role of quorum sensing in antibiotic resistance [62]. A quorum sensing system is a cell density-based intercellular communication system, which utilizes hormone-like compounds referred to as autoinducers to regulate bacterial gene expression [63]. Quorum sensing can regulate multidrug resistance by upregulation of biofilm-associated genes and efflux pump genes [64,65]. Using an integrated proteomics study, Piras et al. [66,67] identified many quorum sensing-related proteins (e.g., LuxS) differentially expressed in multi-drug resistant *E. coli* compared to a control *E. coli* group, suggesting the association between quorum sensing and drug resistance. In *A. baumannii*, the quorum sensing system has also been identified and proved to play a key role in the regulation of bacterial virulence and biofilm formation [68]. The inhibition of the quorum-sensing activity in *A. baumannii* was found able to attenuate biofilm formation and decrease bacterial virulence [69,70], indicating that quorum sensing could be a promising target for developing new strategies against *A. baumannii* infection. More studies will be required to investigate how quorum sensing might be directly associated with antibiotic resistance in *A. baumannii* and how a vaccine targeting the quorum sensing mechanism might be effective against antibiotic resistance.

In addition to the conventional RV prediction, our study has a novel focus on identifying resistance-conferring proteins feasible for vaccine development against drug-resistant pathogens. Targeting vaccines against resistance determinants has been proposed to be a possible effective way to counteract selection pressure for antimicrobial resistance [71,72]. However, there is no report on using RV strategy to predict resistance determinants against antibiotic resistance. Our strategy of RV study of resistance determinants relies on two approaches: (1) Using Vaxign to compare MDR strains vs. three antibiotic-sensitive strains (Figure 1); (2) Analysis of our collection of 84 resistance-conferring proteins from all reported *A. baumannii* strains. We assume that antibody response is the most important against antibiotic resistance. Therefore, our study focused on those OMPs, extracellular proteins, and adhesins, against which antibodies are very effective.

In general, using the first approach, we found 366 proteins that do not exist in any of the three antibiotic-sensitive strains (Figure 1). Out of these 366 proteins, our study found hypothetic outer membrane protein CAM86003.1 and hypothetic protein CAM86739.1, both having high adhesin probabilities. Using the second method, we predicted three extracellular and outer membrane proteins. In addition, we identified 21 periplasmic proteins, among which three have high adhesin probabilities.

RND efflux pumps are organized as a three-component system: a transporter (efflux) protein, located in the inner (cytoplasmic) membrane; a periplasmic accessory protein (also known as a membrane fusion protein (MFP); and an OMP channel, located in the outer membranes of Gram-negative bacteria. In *Campylobacter jejuni*, CmeC is an essential OMP of the CmeABC RND multidrug efflux pump. Zeng et al. discovered that the antibodies of the CmeC peptide inhibited the function of the CmeABC efflux pump and enhanced the susceptibility of *C. jejuni* to natural antimicrobial (bile salts) present in the intestine [73]. Purified recombinant CmeC also stimulated CmeC-specific serum IgG responses via oral vaccination in a chicken model of *C. jejuni* infection; however, the recombinant CmeC vaccination did not confer protection against *C. jejuni* infection [73]. It has also been demonstrated that inhibition of multidrug RND efflux pumps by efflux pump inhibitors (EPIs) is an effective approach to improve the antimicrobial drug susceptibilities of clinical *A. baumannii* isolates [74]. These findings suggest that outer membrane RND efflux pump proteins may be promising vaccine candidates to enhance clinical antibiotic activity and possibly prevent infections by MDR *A. baumannii* strains.

From our analyses of 33 genomes and 84 resistance determinants, three outer membrane RND transporters were identified as vaccine candidates against drug resistance. Our 33-genome analysis identified two RND type efflux pump proteins, CAM85249.1 (CzcC) and CAM85825.1. Since these two proteins were not reported in the literature or databases, they were not included in our original list of 84 resistance determinants. Our study with 84 resistance determinants found one outer membrane RND transporter (WP_000045119.1). To further evaluate their value as vaccine candidates, we used Vaxign to analyze *C. jejuni* CmeC (discussed above) [73] and compared different Vaxign analysis results. Interestingly, *C. jejuni* CmeC [73] was found to have adhesin probability (0.371) less than 0.51, suggesting a lack of the adhesin role [43]. In contrast, CzcC and CAM85825.1 had adhesin probabilities of 0.64 and 0.51, respectively, suggesting both are possible adhesin proteins. Given the importance of adhesins as likely protective antigens, CzcC and CAM85825.1 might be more likely than *C. jejuni* CmeC [73] to stimulate protection against virulent bacterial infection.

Our RV studies on the 33 genomes and 84 resistance determinants found two outer membrane porin (OprD)-like vaccine candidate proteins: CAM88440.1 in strain AYE and WP_000018327.1. OprD promotes the uptake of basic amino acids and small peptides containing these amino acids, and it can also serve as a specific channel for carbapenems by structural homology [75]. Carbapenems are antibiotics for treating infections caused by MDR bacteria. The loss of or reduced OprD confers resistance to carbapenems in *P. aeruginosa* [76,77]. However, Catel-Ferreira et al. demonstrated that the lack of OprD did not affect the susceptibility of *A. baumannii* to treatment with carbapenem antibiotics [78], suggesting that *A. baumannii* OprD is likely not involved in the carbapenem resistance mechanism. More investigation is needed to determine their exact role in antibiotic resistance and potential as vaccine candidates. Among 84 resistance determinants, QnrA (ADB64519.1) is the only predicted extracellular protein. The first discovered QnrA is coded by a 56-kb broad-host range conjugative plasmid, pMG252, that confers an unusual multidrug resistance phenotype, including resistance to quinolones, β-lactams, aminoglycosides, sulphonamides, trimethoprim, and chloramphenicol [79]. QnrA belongs to the pentapeptide repeat family and protects DNA gyrase and type IV topoisomerase from quinolone inhibition. The Qnr determinants have been identified in a series of enterobacterial species and nonenterobacterial Gram-negative species like *P. aeruginosa* and *A. baumannii* worldwide. Many epidemiological surveys show an association between Qnr-like determinants and the β-lactamases with the widest spectrum of activity, i.e., carbapenemases (Ambler class A or class β-lactamases) [80]. In addition to being extracellular, our analysis also found that QnrA has a high antigenicity score. Therefore, a vaccine targeting QnrA is likely to stimulate a strong antibody response against this protein and thus reduce or eliminate the bacterial resistance to many drugs.

β-Lactamases are likely the most critical antibiotic resistance enzymes produced by bacteria to provide multi-resistance to various β-lactam antibiotics. Previous reports show that β-lactamases can be used as vaccine candidates to stimulate protective immunity against antibiotic-resistant pathogens [14,15,81]. Ciofu et al. showed that animals immunized with the AmpC β-lactamase protein could induce strong neutralizing antibody responses and demonstrated a synergistic effect with ceftazidime treatment of resistant *P. aeruginosa* in a rat model of chronic lung infection [14]. This effect could be explained partially by the inactivation of the enzymatic β-lactamase activity by specific antibodies against β-lactamase. Ciofu [15] also found that a rat with chronic lung infection immunized with purified chromosomal β-lactamase showed significantly lower bacterial load and reduced lung pathology compared to non-immunized rats. Zervosen et al. [81] generated a subunit recombinant TEM-1 β-lactamase by insertion of a heat-stable enterotoxin sequence at position 197 of the TEM-1, and immunization of cattle with this hybrid β-lactamase protein resulted in high levels of the anti-TEM IgG in cattle sera that inhibited β-lactamase activity. These results support the feasibility of developing a preventive or therapeutic β-lactamase vaccine that would induce neutralizing antibodies to inhibit the activity of β-lactamase.

Our RV study shows that not all β-lactamases are equally feasible for β-lactamase vaccine development. Specifically, Figure 2 clearly shows the sequence differences and phylogenetic variation among different β-lactamases. In addition, among 39 β-lactamases, we found that all four CTX-M type β-lactamases have the highest adhesin probabilities. CTX-Ms have been detected in at least 26 bacterial species and are widespread not only in humans but also in animals and environments. CTX-M-2, -5, -15 and -43 were reported in *A. baumannii*. In this study, *A. baumannii* CTX-M-2, -5, and -43 enzymes showed preferred adhesion ability >0.51. How these periplasmic β-lactamases possibly become adhesins is unclear. It has been reported that β-lactamases can be released through outer membrane vehicles (OMVs) [47,48]. It is possible that these CTX-M type β-lactamases are released through OMVs and function as bacterial adhesins. Adhesins are usually virulence factors supporting bacterial invasion. Rao et al. also showed that the presence of PER-1 (a class A β-lactamase) is critical to cell adherence [82]. Interestingly, our Vaxign predicted PER-1 as a cytoplasmic protein with an adhesin probability =0.378. Given previous reports of β-lactamases being feasible vaccine candidates [14,15,81] and our Vaxign analysis of the differences among β-lactamases, we would recommend that *A. baumannii* CTX-M-2, -5, and -43 enzymes be better β-lactamase vaccine candidates, which could potentially stimulate strong immune responses that not only reduce antibiotic resistance but also prevent MDR bacterial invasion and infection.

It is possible to develop a combinatorial vaccine that includes a classical type vaccine candidate(s) against bacterial infections and an antibiotic resistance determinant vaccine candidate(s) against drug resistance. Our RV study provides many candidates for such multicomponent vaccine development. The usage of resistance-conferring proteins as antigens in multicomponent vaccines would exert consistent selection against resistance [83]. Furthermore, many antibiotic resistance determinants identified in our RV analysis are also OMPs, extracellular proteins, and adhesins, which are likely to stimulate strong immune responses to serve not only therapeutic but also preventive purposes. These vaccine antigen candidates can be used in different vaccine types such as subunit protein vaccines, epitope peptide vaccines, DNA vaccines, or recombinant vector vaccines.

Reverse vaccinology emphasizes bioinformatics analysis, which is the focus of the current research. It is clear that many valuable results have been identified from our bioinformatics study. It is noted that many published experimental studies [14,22,46,73,81] have experimentally verified many of our predictions. Considering that we did not use these published results ahead of our bioinformatics predictions, these experimental evidences have provided some proof-of-concept evaluations for our analysis. More wet-lab verification studies will definitely be needed in our or others’ future research in order to eventually develop effective and safe vaccines against *A. baumannii* infections, especially those caused by antibiotic-resistant *A. baumannii* strains.

## 4. Materials and Methods

### 4.1. Collection of A. baumannii Genome Sequences and Antibiotic Resistance Determinants

The information for the 33 completed and annotated *A. baumannii* genomes was retrieved from the NCBI database (Available online: http://www.ncbi.nlm.nih.gov/genome/). In addition, 84 *A. baumannii* antibiotic resistance proteins, which cause the resistance of *A. baumannii* strains to different classes of antimicrobial agents, were also retrieved from the ARDB-Antibiotic Resistance Genes Database (Available online: https://ardb.cbcb.umd.edu/) and NCBI Gene and Protein databases.

### 4.2. Vaxign Calculation of Sequence-Derived Features

Thirty-three completed and annotated *A. baumannii* genomes with their NCBI BioProject numbers were used for Vaxign dynamic analysis (Figure 1). Given the BioProject numbers, Vaxign automatically retrieved the protein sequences of the chromosome and any possible plasmid(s) of the *A. baumannii* genome from the BioProject database. For each protein, Vaxign calculated many features including subcellular localization, transmembrane domain prediction, adhesin probability, conservation among different *A. baumannii* strains and homology with human proteins. The programs used in the Vaxign calculation include PSORTb2.0 for subcellular localization prediction [84], TMHMM for transmembrane helix topology analysis [85], and SPAAN for adhesin probability calculation [43]. Vaxign optimizes these programs in a seamless integrative pipeline. After each genome was separately processed, Vaxign then implemented an OrthoMCL program to identify conserved sequences among genomes [86]. The default Blast *E*-value threshold of 10^−5^ was set for OrthoMCL processing [27]. The homology of the antigen candidates to the host (in this study, only human was considered) was tested using Blastp with an *E*-value threshold of 10^−6^.

### 4.3. Vaxign Results Analysis

The final calculated results of the Vaxign pipeline execution were visualized using the Vaxign web program. The Vaxign filtering program was also used to identify proteins that met pre-defined criteria. The Vaxign results were also downloaded to an Excel file and separately analyzed. The Vaxign RV analysis results of the 33 *A. baumannii* genomes are available openly on the Vaxign web page (Available online: http://www.violinet.org/vaxign) for free exploration.

### 4.4. Antigenicity Prediction

Antigenicity scores of potential vaccine candidates were calculated by the VaxiJen v2.0 server. This software uses the z-descriptor composed of multiple physicochemical properties of proteins to predict their antigenicity from FASTA-submitted amino acid sequences using partial least squares discriminant analysis (DA-PLS). The antigens having values more than 0.4 were considered potentially antigenic as described by Doytchinova and Flower [44].

### 4.5. BLAST Analysis and COG Functional Annotation of Predicted Vaccine Candidates

Blastp (Available online: https://blast.ncbi.nlm.nih.gov/Blast.cgi) was used to find the similarity of the potential vaccine candidates to proteins of known function.

The COG protein function assignment was performed using the EggNOG 4.5 server (Available online: http://eggnogdb.embl.de/#/app/home) [87].

### 4.6. PFam Conserved Domain Analysis

The PFam database (Available online: http://pfam.xfam.org/) was used to search the conserved domains present in candidate proteins [88].

## 5. Conclusions

Using 33 complete *A. baumannii* genomes and 84 representative antibiotic resistance determinants, we applied the Vaxign reverse vaccinology pipeline and other bioinformatics methods and identified many vaccine antigen candidates for the rational development of vaccines against *A. baumannii* infections and antibiotic multi-drug resistance. Our study predicted 35 protective antigens that are conserved in all 33 genomes, have no homology with human proteins, have less than 2 transmembrane helices, have high adhesin probabilities, and exist in outer membrane or extracellular locations. These 35 antigens include 11 TonB dependent receptors, 8 porins, 7 efflux pump proteins, and two fimbrial proteins. By comparing the genome sequences of 3 antibiotic-sensitive strains and 30 antibiotic-resistant strains, we identified many vaccine candidates including hypothetic outer membrane adhesin CAM86003.1 and hypothetic adhesin CAM86739.1. By comparatively analyzing 84 resistance determinants and 33-genome sequences, we further identified many feasible anti-resistance vaccine candidates such as three RND type outer membrane efflux pump proteins, one extracellular protein (QnrA), and three CTX-M type β-lactamases. Out of 39 β-lactamases, our study showed that *A. baumannii* CTX-M-2, -5, and -43 enzymes are likely better β-lactamase vaccine candidates than other β-lactamases. To our knowledge, our study is the first to apply reverse vaccinology for systematically predicting vaccine candidates against antibiotic resistance of a microbial pathogen.

## Figures and Tables

**Table 1:** 33 complete *A. baumannii* genomes used in our reverse vaccinology (RV) study.

Strain	NCBI BioProject No.	MDR	Year	Country	Disease/Source	Proteins
ATCC17978	17,477	No	1951	USA	Meningitis	3803
AB307-0294	30,993	No	1994	USA	Bloodstream infection	3363
D1279779	61,919	No	2009	Australia	Bacteremia	3371
A1	269,083	Yes	1982	UK	-	3626
LAC-4	242,902	Yes	1997	USA	Nosocomial outbreak	3633
AYE	28,921	Yes	2001	France	Urinary tract infection	3725
AB0057	21,111	Yes	2004	USA	Bloodstream infection	3669
1656-2	42,153	Yes	2004–2005	Korea	Outbreak strain	3733
ACICU	17827	Yes	2005	Italy	Outbreak strain causing meningitis	3613
MDR-ZJ06	28,333	Yes	2006	China	Ventilator-associated pneumonia	3688
AbH12O-A2	261,783	Yes	2006–2008	Spain	Nosocomial outbreak	3542
AB5075-UW	243,297	Yes	2008	USA	Combatant wound infection	3819
3207	311,558	Yes	2008	Mexico	Bronchoalveolar lavage fluid	3790
D36	294,725	Yes	2008	Australia	-	3848
TCDC-AB0715	62,279	Yes	2007–2009	Taiwan	Bacteremia	3956
AC29	238,628	Yes	2011	Malaysia	Wounds	3555
AC30	173,033	Yes	2011	Malaysia	Wounds	3660
MDR-TJ	52,959	Yes	2012	China	-	3872
TYTH-1	74,551	Yes	2012	Taiwan	Bacteremia	3628
KBN10P02143	291,316	Yes	2012	Korea	Surgical patient pus	3871
BJAB07104	74,421	Yes	2013	China	Bloodstream infection	3754
BJAB0715	74,423	Yes	2013	China	Cerebrospinal fluid	3756
BJAB0868	74,425	Yes	2013	China	Ascites	3689
AB031	256,158	Yes	2014	Canada	Bloodstream infection	3472
AB030	256,157	Yes	2014	Canada	Bloodstream infection	4155
ZW85-1	219,230	Yes	2014	China	Diarrheal patient feces	3544
NCGM237	1466	Yes	2014	Japan	-	3741
XH386	273,343	Yes	2016	China	Pediatric hospital	3942
YU-R612	309,091	Yes	2016	Korea	Sepsis	3900
IOMTU433	3154	Yes	-	Nepal	-	3868
CIP70.10	9585	Yes	-	-	-	3551
R2090	9721	Yes	-	-	-	3526
R2091	12,156	Yes	-	-	-	3567

-: information not found. The list is ordered by the MDR status and then year of isolation or report.

**Table 2:** Categorization of 84 antibiotic resistance determinants collected for this study.

Drug Class	Resistance Mechanism	Protein Category
β-lactams	β-Lactamases	
	Ambler Class A	TEM-1,-19,-116,-128,-193,-194,-195; SHV-1b,-2,-5,-12,-18,-48,-56,-71,-96; CTX-M-2,-5,-15,-43; PER-1; VEB-1,-7;
	Ambler Class B	IMP-1,-4,-5,-8,-19;VIM-1,-2,-11; SIM;NDM-1;
	Ambler Class C	ADC;
	Ambler Class D	OXA-2,-10,-23,-24,-58;
	OMPs	CarO;
	Efflux pumps	RND family: adeC/adeK/oprM, AdeA/AdeI, AdeB, hemolysin D; MATE family efflux transporter: AbeM; MFS family drug transporter: Bcr/CflA
Aminoglycoside	Aminoglycoside-modifying enzymes	Aac(6')-Ib, AacC2, Aac (3)-Ia, Aac(6′)-Ih, Aac(6')-Ik, Aac6-II, Aac(3)-Ia, Aad(2′)-1a, SPH, AphA1-IAB, APH(3′)-VIa;
Quinolones	Quinolone resistance protein	QnrA1;
Tetracyclines	Tetracycline-specific efflux	TETA(A), TETA(G);
Chloramphenicol	chloramphenicol acetyltransferase	chloramphenicol acetyltransferase; chloramphenicol resistance protein;
Sulfanilamide		Sul1;
Tremethoprim	dihydrofolate reductase	DfrA1, DfrA10, DHFRX;
Polymyxin		Polymyxin resistance protein ArnT;

ADC: *Acinetobacter*-derived cephalosporinase; APH: aminoglycoside phosphotransferase; NDM: New Delhi metallo-beta-lactamase; OMPs: outer membrane proteins; OXA: oxacillinase; RND: resistance nodulation cell division; SPH: streptomycin phosphotransferase; TETA: tetracycline resistance protein.

**Table 3:** Predicted *A. baumannii* vaccine candidates based on genome sequence analysis.

Protein Accession	Protein Name	Localization	Adhesin Probability	Length	Antigenecity Score	COG Group	Pfam Domains	Functional Description
**TonB Dependent Receptor Proteins**								
CAM86801.1	putative ferric siderophore receptor	OM	0.639	734	0.60	P	PF00593	TonB dependent receptor
CAM88090.1	putative ferric siderophore receptor	OM	0.612	743	0.67	P	PF00593	TonB dependent receptor
CAM86392.1	putative siderophore receptor	OM	0.558	756	0.59	P	PF00593	TonB dependent receptor
CAM86878.1	putative ferric siderophore receptor	OM	0.600	772	0.61	P	PF00593	TonB dependent receptor
CAM85131.1	putative ferric siderophore receptor	OM	0.595	736	0.67	P	PF00593	TonB dependent receptor
CAM86022.1	putative ferric acinetobactin receptor (bauA)	OM	0.627	767	0.59	P	PF00593	TonB dependent receptor
CAM86399.1	putative OM porin, receptor for Fe(III)-coprogen, Fe(III)-ferrioxamine B and Fe(III)-rhodotrulic acid uptake (FhuE)	OM	0.574	718	0.58	P	PF00593	TonB dependent receptor
CAM87481.1	putative TonB-dependent siderophore receptor precursor	OM	0.531	705	0.70	P	PF00593	TonB dependent receptor
CAM86048.1	putative TonB-dependent receptor	OM	0.566	924	0.59	P	PF00593	TonB dependent receptor
CAM86923.1	putative OM TonB-dependent receptor	OM	0.544	904	0.62	P	PF00593	TonB dependent receptor
CAM85573.1	putative TonB-dependent OM receptor for vitamin B12/cobalamin transport (Btub)	OM	0.644	638	0.67	P	PF00593	TonB dependent receptor
**Fimbria or Pilus Related Proteins**								
CAM87009.1	putative Fimbria adhesin protein	EC	0.710	341	0.68	NU	PF00419	Fimbrial protein
CAM87933.1	putative pilus assembly protein (FilF)	OM	0.677	641	0.73			
**Porin Proteins**								
CAM87753.1	putative OMP	OM	0.606	217	0.75	M	PF00691	OmpA family lipoprotein
CAM88440.1	putative OMP	OM	0.549	438	0.67	M	PF03573	OprD
CAM85599.1	putative glucose-sensitive porin (OprB-like)	OM	0.655	417	0.55	M	PF04966	OprB
CAM86576.1	porin	OM	0.605	439	0.61	M	PF04966	Carbohydrate-selective porin, OprB family
CAM85154.1	conserved, putative exported protein	OM	0.580	255	0.82	S	PF16956	Porin_7 superfamily
CAM85174.1	conserved, putative exported protein	OM	0.599	300	0.72	S	PF16596	Porin_7 Superfamily
CAM85116.1	conserved, putative exported protein	OM	0.584	241	0.80	M	PF03502	Nucleoside-specific channel-forming, Tsx
CAM87023.1	conserved, putative exported protein	OM	0.659	443	0.67	S	PF 02530	OM_channels
**Efflux Related Proteins**								
CAM85249.1	cation efflux system protein (CzcC)	OM	0.518	471	0.64	M	PF02321	OM efflux protein
CAM85825.1	conserved, putative exported protein	OM	0.625	499	0.51	M	PF02321	OM efflux protein
CAM88576.1	polysaccharide export protein	OM	0.530	366	0.46	M	PF02563	Polysaccharide biosynthesis/export protein
CAM87663.1	conserved, putative exported protein	OM	0.608	398	0.71	S		
CAM85361.1	toluene tolerance efflux transporter (ABC superfamily, peri-bind)	EC	0.662	226	0.74	Q	PF02470	MlaD protein
CAM86485.1	conserved, putative exported protein	EC	0.533	294	0.69	S	PF 16331	TolA binding protein trimerization
CAM87843.1	conserved, putative exported protein	EC	0.744	715	0.75	S		
**Other Putative OM Proteins Or Lipoproteins**								
CAM86480.1	putative OM protein	OM	0.541	841	0.63	M	PF01103	Bac_surface_Ag
CAM87743.1	OM lipoprotein	OM	0.536	132	0.42	M	PF04355	SmpA_OmlA
CAM87612.1	putative lipoprotein	OM	0.513	159	0.68	MP	PF04170	copper resistance protein NlpE
**Other Putative Extracellular Proteins**								
CAM85672.1	putative fatty acid transport protein	EC	0.660	476	0.64	M	PF03349	
CAM88107.1	putative phosphatase; alkaline phosphatase	EC	0.561	726	0.49	S	PF05787	unknown function
CAM85335.1	putative alkaline protease	EC	0.551	461	0.52	O	PF00082	Peptidase_S8
CAM85336.1	conserved, putative signal peptide	EC	0.570	143	0.44			

In column COG group, M: Cell wall/membrane/envelope biogenesis; P: Inorganic ion transport and metabolism; O: Post-translational modification; Q: Secondary metabolite biosynthesis, transport, and catabolism; NU: Cell motility, Intracellular trafficking, secretion, and vesicular transport; MP: Cell wall/membrane/envelope biogenesis, Inorganic ion transport and metabolism; S: Function unknown; OM: Outer membrane; EC: Extracellular.

**Table 4:** Predicted *A. baumannii* vaccine candidates based on sequence analysis of antibiotic resistance determinants.

Accession Number	Resistance Protein Name	Protein Length	Localization	Adhesion Probability	Antigenicity Score	COG Group	Pfam Domains	Functional Description
ADB64519.1	QnrA1	218	EC	0.330	0.58	S	PF00805 PF13599	Pentapeptide repeat protein
WP_000045119.1	adeC/adeK/oprM family multidrug efflux complex outer membrane factor	465	OM	0.339	0.62	M	PF02321	RND efflux system, outer membrane lipoprotein
WP_000018327.1	OprD-like protein	469	OM	0.533	0.70	M	PF03573	OprD super family Outer membrane porin
AAL68825.1	CTX-M-5	276	PP	0.598	0.47	V	PF13354	β-lactamase
AAZ14955.1	CTX-M-43	291	PP	0.548	0.43	V	PF13354	β-lactamase
BAD34451.1	CTX-M-2	291	PP	0.537	0.42	V	PF13354	β-lactamase
AEQ20897.1	CTX-M15	291	PP	0.427	0.44	V	PF13354	β-lactamase
ACJ61335.1	RTG-4	298	PP	0.384	0.42	V	PF13354	β-lactamase
AFA35105.1	ADC	383	PP	0.367	0.34	V	PF00144	β-lactamase
ACO56763.1	VEB-7	299	PP	0.354	0.54	V	PF13354	β-lactamase
AMB18971.1	TEM-116	291	PP	0.251	0.50	V	PF13354	β-lactamase
AFN21551.1	TEM-19	286	PP	0.234	0.47	V	PF13354	β-lactamase
AAQ57123.1	TEM-128	286	PP	0.233	0.47	V	PF13354	β-lactamase
AGW28875.1	TEM-1	286	PP	0.226	0.48	V	PF13354	β-lactamase
AFC75524.1	TEM 194	286	PP	0.212	0.49	V	PF13354	β-lactamase
AFC75525.1	TEM 195	286	PP	0.212	0.48	V	PF13354	β-lactamase
AAQ55480.1	SHV-56	286	PP	0.210	0.46	V	PF13354	β-lactamase
AFC75523.1	TEM 193	286	PP	0.207	0.47	V	PF13354	β-lactamase
ACG63555.1	SHV-18	286	PP	0.203	0.43	V	PF13354	β-lactamase
AAV38100.1	SHV-1b	286	PP	0.194	0.46	V	PF13354	β-lactamase
AAP20889.1	SHV-12	286	PP	0.185	0.45	V	PF13354	β-lactamase
ABC25482.1	SHV-71	286	PP	0.177	0.45	V	PF13354	β-lactamase
AAP20890.1	SHV-48	286	PP	0.176	0.46	V	PF13354	β-lactamase
ABN49112.1	SHV-96	286	PP	0.165	0.48	V	PF13354	β-lactamase

In column COG group, M: Cell wall/membrane/envelope biogenesis; V: Defense mechanisms; S: Function unknown. OM: Outer membrane location; EC: Extracellular location; PP: Periplasmic location.

## Supplementary Materials

Supplementary materials can be found at www.mdpi.com/1422-0067/18/2/458/s1.
